# The prevalence of human immunodeficiency and of hepatitis B viral infections is not increased in patients with sickle cell disease in Tanzania

**DOI:** 10.1186/s12879-021-06726-z

**Published:** 2021-09-30

**Authors:** Grace Shayo, Irene Makundi, Lucio Luzzatto

**Affiliations:** 1 Department of Internal Medicine, Muhimbili University of Health and Allied Sciences, P.O.Box 65001, Dar es Salaam, Tanzania; 2 Department of Internal Medicine, Muhimbili National Hospital, P.O.Box 65000, Dar es Salaam, Tanzania; 3 Department of Hematology and Blood Transfusion, Muhimbili University of Health and Allied Sciences, P.O.Box 65001, Dar es Salaam, Tanzania

**Keywords:** Sickle cell disease, HIV, HBV, Anemia, Dar es Salaam

## Abstract

**Background:**

Tanzania ranks as the fourth country in the world with respect to the number of sickle cell disease (SCD) births; it is also endemic to the human immunodeficiency virus (HIV) and the hepatitis B virus (HBV). This study was done to determine the prevalence of HIV and HBV infections among SCD patients in Dar es Salaam, Tanzania.

**Methods:**

A multicenter hospital-based descriptive cross sectional study was carried out among participants aged ≥ 16 years with a proven diagnosis of SCD. Socio-demographic and clinical data were recorded. Blood samples were drawn for HIV and HBV diagnosis. All categorical variables were summarized into frequencies.

**Results:**

There were 185/325 (56.9 %) females. The mean age (SD) was 23.0 ± 7.5 years. The prevalence of HIV was 1.8 %; the prevalence of HBV was 1.2 %.

**Conclusions:**

The prevalence of both HIV and HBV in SCD patients is no greater than in the general population of Dar es Salaam or Tanzania. For associations, a large study would be needed. From a detailed blood transfusion history of SCD patients we found no evidence that HIV or HBV infection was transmitted through blood transfusion.

## Background

It is estimated that 300,000 children are born annually in the world with sickle cell disease (SCD). An estimated 75 % of SCD affected children live in sub-Saharan Africa [1, 2]. Tanzania is ranked as the fourth country globally with the highest number of SCD births after Nigeria, Democratic Republic of Congo and India [2, 3].

Improvements in the care of SCD have increased the life span of these patients. Thus, there are now more SCD children who grow into adulthood and face other health risks, including infections. Human immunodeficiency virus (HIV) and hepatitis B virus (HBV) infections are transmissible diseases that may add a burden to SCD patients. It is estimated that nearly one adult in every 25 is living with HIV in Sub-Saharan Africa, that thus accounts for nearly two-thirds of the people living with HIV worldwide [4]. In 2017 Dar es Salaam had a 4.7 % prevalence of HIV infection while the prevalence in Tanzania as a country was 5.1 % [5]. Globally, HBV prevalence was estimated to be 3.5 % in 2015 resulting in an estimated 887,000 hepatitis B-related deaths, mostly from complications such as cirrhosis and hepatocellular carcinoma. Africa and Western Pacific regions accounted for 68 % of those infected with HBV [6]. Tanzania is among the countries with the highest HBV infection rates. A prevalence of 6 % was reported in 1998 among the general population of the largest city in the country, Dar es Salaam [7]. Another study carried out in 2004–2005 among blood donors in the same city revealed a prevalence of 8.8 % for HBV and 3.8 % for HIV infection [8].

HIV and HBV have similar modes of transmission: therefore, co-infection is common [9, 10]; and HIV co-infected patients have an increased risk of developing chronic HBV [11]. HIV infection of an SCD patient may pose special challenges in antiretroviral therapy (ART): as anemia is a contra-indication for certain drugs, as illustrated by a case report from Kenya [12].

SCD has been thought to be protective against acquiring HIV [13, 14], and it may slow down the progression of HIV disease [15] through host factors such as functional asplenia, or the use of hydroxyurea, that may be synergistic with some reverse transcriptase inhibitors [16, 17]. On the other hand, SCD patients with HIV infection tend to have more complicated hospitalization than those without HIV infection [18]. SCD patients are highly susceptible to infection with encapsulated organisms, and this susceptibility may be increased by concomitant HIV infection [19, 20].

Blood transfusion has been reported to be a risk factor for HIV acquisition in SCD patients in studies conducted in some African countries such as Democratic Republic of Congo [21], Nigeria [22] and Cameroon [23]. Unsafe blood transfusion is also a risk factor for HBV infection. In the USA, however, it was found that among adults with SCD the frequency of HIV was 1.5 %, compared to 3.3 % in African–Americans without SCD, suggesting that blood transfusion was not a risk factor.

This study was conducted to investigate the burden of HIV and HBV among SCD patients receiving SCD care in Dar es Salaam, Tanzania. This study was previously presented as an abstract at the 7th MUHAS Scientific Conference on June 27–28, 2019.

## Methods

### Design and setting of the study

A cross sectional multisite hospital-based study was conducted from August 2018 to January 2019 among sickle cell patients attending clinics in Dar es Salaam. Dar es Salaam is the largest city in Tanzania and has five municipal districts and an estimated population of 4,364,541 as per the 2012 National census [24]. The study participants were obtained from four study sites, one being a tertiary hospital (Muhimbili National Hospital) and three regional referral hospitals (Amana, Temeke and Mwananyamala). Sickle cell patients at Muhimbili National Hospital (MNH) received care in the department of Haematology and blood transfusion while those in the three regional hospitals received care in departments of internal medicine. All clinics ran once a week and received an average of 15 adult patients per week.

### Study population

All consenting SCD patients aged 16 years or older who attended sickle cell clinics in Dar es Salaam were consecutively enrolled in the study. None of the patients was excluded.

### Data collection procedure

A structured interview was done with all patients who consented to participate in the study. Independent variables collected were socio-demographic characteristics including age, sex, marital status, level of education, residency and occupation. Other risks of interests were condom misuse, multiple sexual partners, tattooing, piercing, intravenous drug abuse, history of blood transfusion, hospital admission, surgery, tooth extraction, sexual vulnerability behaviours such as alcohol use and forced sexual intercourse. The value of the last measured hemoglobin (HB) level was obtained from the SCD card. The HB was considered valid if it was done within 1 month of data collection.

Four milliliters of blood was drawn aseptically from a vein on the cubital fossa and put into an empty sterile red stoppered vacutainer for HIV and HBV screening. At the study sites samples were stored in cool boxes before being transferred to MUHAS Hematology Clinical and Research Laboratory at the end of each clinic day. Upon reaching the laboratory, samples were centrifuged, sera taken and tested for HBV surface antigen and HIV serology. HIV diagnosis was in accordance with Tanzanian algorithm [25] whereby SD Bioline HIV-1/2 3.0 (Standard Diagnostics, Inc, Republic of Korea) was done first. Negative results underwent no additional testing. Positive samples were retested using Determine™ hiv-1/2 (Abbots Laboratories,

Illinois, USA). Samples reactive to both tests were considered positive for IgG anti HIV antibodies. Positive tests By SD Bioline that tested negative by Determine™ hiv-1/2 were subjected to a third test, Uni-Gold™ HIV (Trinity Biotech Plc, Bray, Co. Wicklow, Ireland) and were considered negative if they tested negative or positive if they tested positive by Uni-Gold™.

Hepatitis B surface antigen was detected by the Onsite HBsAg rapid test by CTK Biotech, Inc, San Diego, USA. The test is a lateral flow chromatographic immunoassay for the qualitative detection of HBsAg in human serum or plasma. The test has a sensitivity and specificity of approximately 100 % and 100 % respectively when performed according to the instructions of the manufacturer. Negative results underwent no additional testing. Positive results were confirmed by ELISA using Murex HBsAg version 3 test kits manufactured by DiaSorin S.p.A, United Kingdom.

### Statistical analysis

Data analysis was done using SPSS software version 20.0. Categorical variables were summarized into frequencies and percentage. Continuous variables were summarized into mean and standard deviation. No associations were calculated between variables and HIV or HBV infections due to small numbers of patients with these infections.

## Results

We tested 185 (56.9 %) females and 140 males with SCD (see Table 1). The mean age (± SD) was 23.0 ± 7.5 years, ranging from 16 to 52 years; 71.1 % were in the age group < 26 years. The majority (85.5 %) of the subjects had never married; 72.3 % had secondary school level education; 91.1 % had previous hospital admission, and the most frequent reason for admission in 56.1 % was a vaso-occlusive crisis. Only 18.8 % subjects were using hydroxyurea (HU) (Table 1). The mean recorded hemoglobin level (± SD) for the 325 study subjects was 7.4 (± 1.6) g/dl, (not shown in Table 1).

Of the 325 study participants, 6 (1.8 %; 95 % CI: 0.7–4.0 %) were HIV-infected; 4 (1.2 %; 95 % CI: 0.3–3.1 %) were infected with HBV. None of the study participants was HIV and HBV co-infected (not shown in Table 1).

All HIV and HBV-infected patients had been hospitalized and had received blood transfusion: the same was true for most of those who were not infected. Of the HIV infected patients none was on HU treatment; of the HBV-infected patients 2 were on HU. Of the 6 HIV-infected patients, 3 were sexually active; 3 had multiple sexual partners, and 2 of them used condoms. Of the 4 HBV-infected patients 3 were sexually active, and 3 reported a history of multiple sexual partners; only one reported using condom during the last sexual act, (Table 1).

**Table 1:** HIV and HBV status in relation to socio-demographic, clinical and lifestyle data in 325 patients with sickle cell disease

Characteristic		Total Number	HIV Positive Number(%)	HBV Positive Number (%)
Sex	Male	140	2 (1.4)	2 (1.4)
	Female	185	4 (2.2)	2 (1.1)
Age	< 26	231	3 (1.3)	1 (0.4)
	26+	94	3 (3.2)	3 (3.2)
Marital status	Never married	278	4 (1.4)	3 (1.1)
	Ever married	47	2 (4.3)	1 (2.1)
Education	Primary or lower	89	3 (3.4)	4 (4.5)
	Secondary or higher	236	3 (1.3)	0 (0)
Occupation	Unemployed or student	221	4 (1.8)	1 (0.5)
	Employed	104	2 (1.9)	3 (2.9)
Previous hospital admission	Yes	296	6 (2)	4 (1.4)
	No	29	0 (0)	0 (0)
Reason for last admission (N = 296)	Painful crisis	166	0 (0.0)	0 (0)
	Anemia	85	3 (3.6)	1 (1.2)
	ACS	5	0 (0.0)	0 (0)
	Others*	40	3 (7.5)	3 (7.5)
History of BT	Yes	243	6 (2.5)	4 (1.7)
	No	82	0 (0.0)	0 (0)
Number of BT units (N = 243)	1 unit	84	2 (2.4)	1 (1.2)
	2–5units	125	1 (0.8)	2 (1,6)
	> 5 units	34	3 (8.8)	1 (2.9)
Hydroxyurea use	Yes	61	0 (0)	2 (3.3)
	No	264	6 (2.3)	2 (0.8)
Sexually active	Yes	185	3 (1.6)	3 (1.6)
	No	140	3 (2.1)	1 (0.7)
Condom use (N = 185)	Yes	67	2 ( (3.0)	1 (1.5)
	No	118	1 (0.8)	2 (1.7)
Multiple sexual partners (N = 185)	Yes	68	3 (4.4)	3 (4.4)
	No	117	3 (2.6)	1 (0.9)
Tooth extraction	Yes	189	4 (2.1)	2 (1.1)
	No	136	2 (1.5)	2 (1.5)
Tattooing	Yes	2	0 (0)	0 (0)
	No	323	6 (1.9)	4 (1.2)

*Others = stroke, surgeries and obstetric reasons, *ACS*  acute chest syndrome, *BT*  blood transfusion

## Discussion

We have studied an adult population with a mean age of 23.0 ± 7.5 years, with a balanced gender ratio. Compared to previous surveys, it is interesting that a majority had secondary or higher-level education, thanks to the government policy to build secondary schools in each ward in Tanzania (2010), and following the introduction of free secondary education in 2015. The patient population we have studied have clearly benefited from these policies.

In this study the HIV prevalence of 1.8 % was lower than that reported in 2016–2017 in the general population of Dar es Salaam, which was 4.7 % [26]. The HIV prevalence was also lower than that observed in a similar study among SCD children in Tanzania, that was 4.4 % (unpublished data by Kamaria Kassim et al.). In studies carried out in other African countries endemic for HIV, frequencies have been higher: 5.6 % in Cameroon, 11.3 % in the Democratic Republic of Congo and 5.0 % in Togo [21, 23]: these studies had included both children and adults, whereas ours was on adults only. It is possible that some patients may have died during childhood, especially if they had perinatally acquired infection. On the other hand, our data may reflect the gradual decline in HIV prevalence that has been observed in Tanzania over recent years.

The prevalence of HBV in the present study was higher than that observed in children with SCD in the year 2010, which was 0.6 % (Kamaria Kassim et al., unpublished data). The difference could be due to cumulative exposure to the hepatitis B virus during life time, thus reflecting increasing prevalence with increasing age. Most important, vaccination against Hepatitis B was introduced in the Extended Programme for Immunization (EPI) in Tanzania in 2002, and most of our participants were born before then. On the other hand, our findings are similar to those in SCD patients in Brazil (3.1 %) [27]; in contrast, the HBV prevalence was as high as 10 % among SCD patients in the Democratic Republic of Congo (29).

Although co-infection with both HIV and HBV has been observed in Tanzania among blood donors [8] and adult HIV patients [30], we have not found it in our SCD patients, as in a similar study from Nigeria [29]. This is not surprising since, from the prevalence rates reported above, the a priori probability of co-infection can be estimated at 0.022 % (about 2 in 10,000); in addition, we must consider that this combination might increase mortality in patients with SCD.

Blood transfusion can of course tramsmit blood borne infections, if blood units are not properly screened. In our entire patient population 75 % had received at least one blood transfusion: this included all 6 HIV-infected and all 4 HBV infected patients. However, out of a total of 243 transfused patients, 233 (95.9 %) remained HIV-negative and HBV-negative. This is rather re-assuring: it indicates that the screenings that are obligatory for blood donors are reliable.

### Study limitation

The present study was not powered to determine associations between the studied variables and the HIV or HBV viral infection.

## Conclusion

The findings of this study suggest that the prevalence of HIV and HBV infection among SCD patients aged 16 years or older is no higher than the prevalence in the general population of Dar es Salaam. For the few SCD patients who are either HIV or HBV infected we do not know the source of infection; there is no reason to suspect that they had been infected via blood transfusion.

## Data Availability

The data set for this study can be found from the corresponding author upon reasonable request.

## Abbreviations

ART: Antiretroviral therapy
ELISA: Enzyme linked immunosorbant assay
EPI: Extended Programme for Immunization
HB: Hemoglobin
HBsAg: Hepatitis B surface antigen
HBV: Hepatitis B virus
HIV: Human immunodeficiency syndrome
MHN: Muhimbili National Hospital
MUHAS: Muhimbili University of Health and Allied Sciences
SCD: Sickle cell disease
SD: Standard deviation
SPSS: Statistical package for social sciences
USA: United States of America
